# A Novel Automated RGB-D Sensor-Based Measurement of Voluntary Items of the Fugl-Meyer Assessment for Upper Extremity: A Feasibility Study

**DOI:** 10.3390/brainsci12101380

**Published:** 2022-10-12

**Authors:** Yue Li, Chong Li, Xiaokang Shu, Xinjun Sheng, Jie Jia, Xiangyang Zhu

**Affiliations:** 1 State Key Laboratory of Machanical System and Vibration, Shanghai Jiao Tong University, Shanghai 200040, China; 2 Department of Rehabilitation Medicine, Huashan Hospital, Fudan University, Shanghai 200040, China

**Keywords:** stroke rehabilitation, upper limb, automated system, motor function assessment, Fugl-Meyer Assessment

## Abstract

Motor function assessment is essential for post-stroke rehabilitation, while the requirement for professional therapists’ participation in current clinical assessment limits its availability to most patients. By means of sensors that collect the motion data and algorithms that conduct assessment based on such data, an automated system can be built to optimize the assessment process, benefiting both patients and therapists. To this end, this paper proposed an automated Fugl-Meyer Assessment (FMA) upper extremity system covering all 30 voluntary items of the scale. RGBD sensors, together with force sensing resistor sensors were used to collect the patients’ motion information. Meanwhile, both machine learning and rule-based logic classification were jointly employed for assessment scoring. Clinical validation on 20 hemiparetic stroke patients suggests that this system is able to generate reliable FMA scores. There is an extremely high correlation coefficient (r = 0.981, *p* < 0.01) with that yielded by an experienced therapist. This study offers guidance and feasible solutions to a complete and independent automated assessment system.

## 1. Introduction

Stroke is one of the leading causes of motor function impairment worldwide, and 30% to 66% of post-stroke hemiplegic patients suffer from permanent upper limb deficits [1]. Restoring upper limb motor function through rehabilitation can considerably improve patients’ lives.

As a crucial step in the stroke rehabilitation process [2], assessment contributes much to rehabilitation training guidance and patients’ self-confidence enhancement. Traditional clinical assessment relies on the professional therapist observing the patient’s behavior based on various scales. According to the International Classification of Functioning, Disability and Health (ICF), these scales can be divided into three main categories [3]: body functions such as Fugl-Meyer Assessment (FMA) and Motor Status Score (MSS), activity ability such as Wolf Motor Function Test (WMFT) and Arm Motor Ability Test (AMAT), and participation such as Stroke Impact Scale (SIS) and EuroQol Quality of Life Scale (QLS). Among them, FMA [4] is probably the most widely used one in both clinical and research applications [5]. It has excellent intra-rater and inter-rater reliability and construct validity [6,7], and is sensitive to change [8]. The FMA upper extremity (FMA-UE) section (Table 1) consists of 33 items, and each item is scored on a 3-point ordinal scale from 0 to 2.

However, the requirement of therapist participation substantially limits the implementation of the assessment. As for patients, especially discharged patients, scientific assessment is not readily available. For therapists, it is time-consuming and laborious. Consequently, automated scale evaluation systems are proposed to solve these problems.

A few studies on automated evaluation of the upper extremity motor function have been conducted using various sensors. Wearable sensors, including inertial measurement units (IMUs) (or only accelerometers) [9,10], flex sensors, and their combinations [11], have been heavily applied to automate FMA or WMFT [12]. They are portable and accurate. Nevertheless, these sensors are inconvenient for patients to wear and the preparation can take a long time, especially when using the glove sensor to track hand motion. Electromyography (EMG) is a bioelectric signal that reflects neuromuscular behaviors, which is of great significance for understanding motor function levels and guiding rehabilitation [13]. By using EMG, both longitudinal [14] and cross-sectional [15] assessments can be implemented from a more physiological perspective. The multi-camera-based optoelectronic system, such as Vicon (Vicon Motion System, USA) and Impulse (Phase Space, USA) [16], is another commonly used sensor with acute precision for automated assessment system construction. However, their high price and complicated operation limit their use in the laboratory.

Compared with the three kinds of sensors mentioned above, the depth camera, such as Kinect (Microsoft, Redmond, WA, USA), RealSense (Intel, Santa Clara, CA, USA), and Leap Motion (Leap Motion Inc., San Francisco, CA, USA), has the advantages of low cost, convenient installation, and high comfort. These advantages, together with acceptable precision, promote their widespread use in rehabilitation. Kim et al. [17] automated 13 FMA-UE items using Kinect. Bai et al. [18] fused Kinect One and a posture sensor to evaluate 15 FMA items as well as the reachable workspace area. Fang et al. [19] proposed a novel hand assessment framework compliant with Swanson impairment evaluation and FMA based on Leap Motion. Further, Lee et al. [20] combined Kinect v2 and FSRs and achieved the automated evaluation of 26 FMA-UE items. It is worth noting that most of these systems merely consider part of the scale: some aim to evaluate the shoulder and elbow joints [17,18], and others focus on assessing hand function [19,21]. The incompleteness diminishes their practical value. In other words, there is still much room for improvement in these systems for application in independent clinical evaluation.

Aiming to achieve complete, independent, and low-cost assessment without therapists’ involvement, this paper proposed an automated evaluation system covering all voluntary FMA-UE items, using no body-worn sensors. Two RGBD cameras (An RGBD camera is a type of depth camera that provides both depth (D) and color (RGB) data) and several force-sensing resistors were included. Both machine learning and rule-based logic classification were applied for score prediction according to different categories. The feasibility of the proposed system was demonstrated with data from 20 hemiparetic stroke patients.

## 2. Materials and Methods

### 2.1. Participants

Applying the two-stage sample size approximation method for the Pearson correlation [22], with the estimated correlation coefficient $\tilde{r}=0.9$, Fisher confidence interval ω=0.3, and significance level α=0.01 as parameters, a correct sample size n=20 can be obtained. Therefore, twenty stroke patients (fifteen males, five females; age: 58.95 ± 10.58 years) from the Rehabilitation Medicine Department of Huashan Hospital were recruited for this study. The inclusion criteria for participation in the study were: (1) age between 21 and 75 years old; (2) diagnosis of unilateral hemiplegia caused by ischemic or hemorrhagic stroke; (3) absence of apraxia and severe medical complications (including shoulder pain); (4) with no serious cognitive impairment and able to understand and follow instructions (Mini-Mental State Examination (MMSE) score > 20 [23]). The study was pre-approved by the Huashan Hospital Institutional Review Board (KY2018-248) and registered at the Chinese Clinical Trial Registry (ChiCTR1800017568). All participants were provided with and signed the informed consent prior to the experiment.

### 2.2. System Design

#### 2.2.1. FMA Items

The complete scale of FMA-UE is shown in Table 1. All these FMA items except the three reflex ones (item 1, 2, and 18 filled with grey) requiring external forces, were covered in this automated system. It has been suggested that the three reflex items contribute little to volitional movement ability measurement and can be excluded from the FMA-UE evaluation [24,25]. Based on assessment joints and execution actions, the FMA-UE can be divided into four categories: Shoulder/Elbow, Wrist/Hand, Grasp, and Coordination/Speed. Overall, 30 FMA items (33 in total) were implemented in this automated system with 17 motion tasks. According to the scale instruction, item 3–11 only involve one movement, and so do item 24–25, 31–33. For convenience, item 13 and 16 were combined into one action: shoulder flexion 180°. Besides, several minor adjustments have been made in view of the actual application. Item 14 and 17 were broken down into two movements respectively: shoulder/elbow moving to the initial position and forearm pronation/supination at this position. Item 19 and 20 were combined into one action because the automated system could not provide resistance to wrists, and so were item 21 and 22. Each of the rest items corresponded to one indicated action.

#### 2.2.2. Hardware and Software

**Figure 1:**
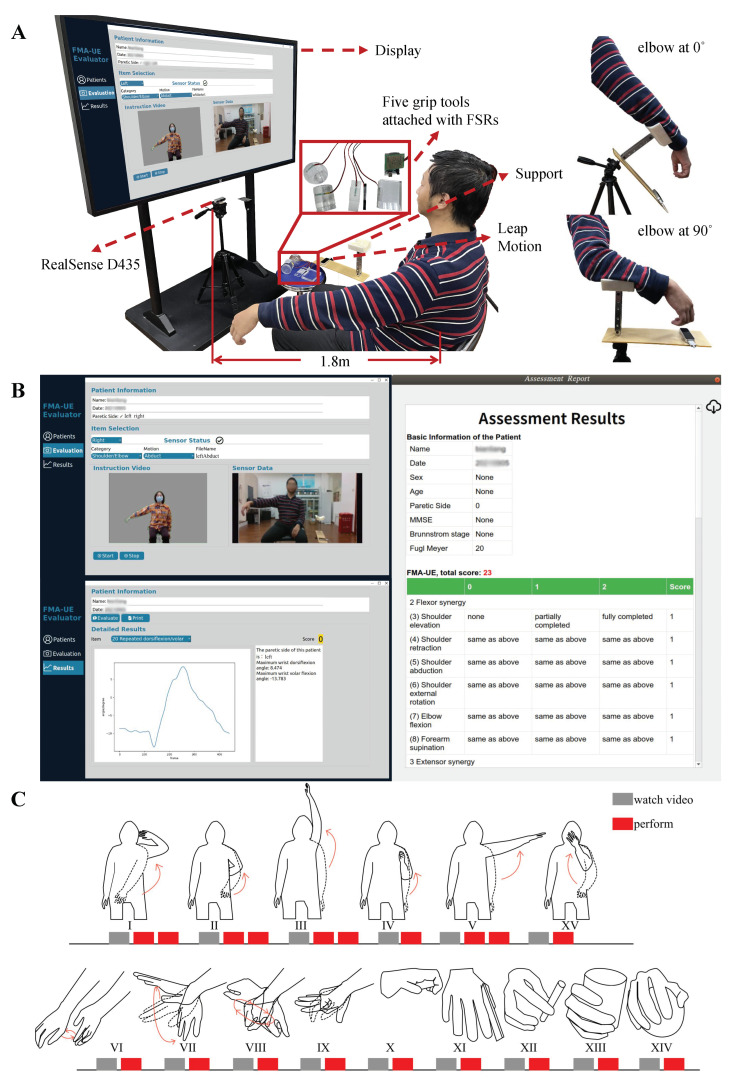
(**A**) The hardware setup and scene of the experiment. On the right are the two initial positions for the Wrist/Hand part. (**B**) The graphical user interface (GUI) of the assessment system. (**C**) The experimental protocol.

The sensors used by the system included a RealSense D435, a Leap Motion, and Force Sensitive Resistors (FSRs) (Interlink Electronics, Westlake Village, CA, USA). The hardware layout is shown in Figure 1A.

**Figure 2:**
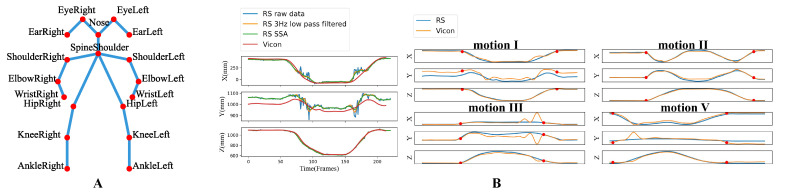
(**A**) 18-joint skeleton of RealSenseD435. (**B**) The 3D coordinates of the wrist joint in the Shoulder/Elbow part motions. Left: The curves of raw RealSense (RS) data, data processed by two denoising methods, and Vicon data of motion I. Right: The SSA-filtered RS data and the Vicon data of four motions, and the red dots mark the motion start and end time.

RealSense D435, a mainstream depth camera having the potential to measure health outcomes [26], was applied to record the movement of the Shoulder/Elbow and Coordination/Speed parts. In this system, RealSense was positioned on a tripod in front of the patient, and data streams were captured at 30 frames per second (fps). The coordinates of 18 joints (Figure 2A) could be extracted from raw data based on skeleton tracking SDK. Leap Motion controller is a depth-sensing camera designed for tracking hand and finger motion at up to 200 fps, which was mainly used in the Wrist/Hand part. At the same time, a support mechanism was specially designed, aiming to fix Leap Motion and human arm comfortably and keep their relative position. The support was placed on the other tripod to achieve the change in pitch angle (Figure 1A). In order to quantitatively measure the interaction force between the hand and the object in the Grasp part, five FSRs were respectively attached to five corresponding grip tools [20]. The detailed information of the raw data collected by the above sensors is summarized in Table 2.

The automatic administration is also a critical aspect of automated evaluation systems [27], which is mainly embodied in the interaction with the user. Therefore, user-friendly software was also developed (Figure 1B). It has the function of patient information register, data collection, data analysis, and results generation. In the data collection interface (left top of Figure 1B), when an evaluation action is selected, the corresponding instruction video will be played on the left, and the real-time sensor data will be displayed on the right. In the results generation interface (left bottom of Figure 1B), the scores and vital kinematic features of single items are available. Finally, a complete FMA-UE report (right of Figure 1B) from this automated system can be viewed and saved.

#### 2.2.3. Experimental Protocol

As shown in the experimental scene in Figure 1A, the subject sat in a chair facing a display. For each movement, an instruction video was played first, and the subject was asked to try to perform the demonstrated action without assistance (Figure 1C). The four Shoulder/Elbow part motions (I, II, III, V) were performed twice. All other motions were performed once on the less-affected side and paretic sides respectively. At the same time, one experienced therapist also participated to observe and rate each FMA item according to the consistency between patients’ performance and the scale instructions.

### 2.3. Assessment

#### 2.3.1. Data Analysis Procedure

**Figure 3:**
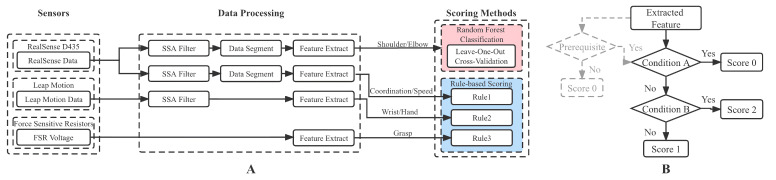
(**A**) Flow chart of data analysis. SSA: singular spectrum analysis. (**B**) The general IF-ELSEIF-ELSE logic model of scoring rules, where the gray part indicates that several items require prerequisites.

Figure 3A depicts the data analysis procedure of the proposed system, in which the extracted features are listed in Table 3. The data from sensors were first preprocessed, and then specific features were extracted. These features served as inputs to the scoring method. Different scoring methods were applied to different categories. The Shoulder/Elbow part was scored using random forest (RF) classification, an effective machine learning algorithm for estimating scale scores from kinematic features [28,29]. It has the advantages of non-parametric nature, feature importance evaluation capability, and high classification accuracy [30]. The other three were scored using rule-based (RB) logical classification, because the movement evaluation criteria are explicit and concise, suitable to directly abstract logical rules with interpretability for scoring. Meanwhile, it was also found that the RF classification could not achieve better results. The following two sections will separately introduce the automated evaluation process of the Shoulder/Elbow part and the other three parts in detail.

#### 2.3.2. The Shoulder/Elbow Part

The RealSense Data consisted of a time series of the 18-key-point 3D positions. Regarding the data with high confidence collected by Vicon as the baseline, it could be found that the raw RealSense data were contaminated by noise (Figure 2B-left). Two algorithms, low pass filter and singular spectrum analysis (SSA) [31], have been compared to attenuate the influence of noise. Finally, SSA was selected because of its stronger smoothing effect. The main steps of SSA include embedding, singular value decomposition, grouping, and reconstruction. It decomposes diverse components (trend, periodic components, noise, etc.) from the initial time series. Selecting the top *k* components with the largest contribution for reconstruction can achieve the purpose of denoising. Taking the wrist joint in motion I as an example, it can be seen from Figure 2B-left that the data filtered by SSA is highly consistent with the Vicon data. Next, the start time and end time were automatically detected by marking the first and the last frames with higher endpoint velocities than the average one. The preprocessed data (i.e., denoised and segmented data) of the wrist joint are shown in Figure 2B-right, where the red dots represent the motion segmentation points. The wrist joint position offsets (mean Euclid distance between the corresponding joints in each time frame) for the four motions (I, II, III, V) were 54, 43, 138, 150 mm, respectively, with a mean offset of 96mm, which was acceptable and close to that of Kinect V2 (72 mm) [32]. Therefore, the RealSense data processed by SSA could achieve high precision with limited data acquisition conditions.

Before the scoring method was applied, 36 kinematic features in Table 3 were calculated to describe the properties of the preprocessed data, which could be divided into three aspects [33]: endpoint kinematics (6 features, e.g., path length, velocity, smoothness [34]), angular kinematics (24 features, e.g., angles and angular velocity of four degrees of freedoms (DoFs)) and other kinematics (6 features, e.g., inter-joint coordination index (IJCI) [35], time, trunk compensation [36]).The detailed calculation of these features are elaborated in Appendix A. 13 RF classifiers, respectively corresponding to 13 items, were trained, for item 3–13,15,16 (Table 1). The features stated above were used as input and the scores evaluated by one experienced therapist were used as labels.

Comprehensively considering the smoothing effect and computation time, the two main parameters of the SSA filter, the window length *L* and the reconstruction subsequence number *k*, were set to 15 and 2, respectively. Additionally, the two main parameters of the RF classifier, the number of trees *n* and the number of features *f*, were set to 100 and 6. The parameter *n* was selected by five-fold cross validation. A sequence of increasing values was applied until the results tended to stabilize.

#### 2.3.3. The Other Three Parts

For the Coordination/Speed part, the parameter *L* of the SSA algorithm was slightly adjusted so as to avoid removing motion details and maintain the tremor information for item 31. For the Wrist/Hand part, the Leap Motion Data was composed of hand bone vectors and some variables (palm orientation, hand opening, closing degree, etc.) generated by Leap Motion SDK. Data were also first preprocessed using an SSA filter. Then specific features listed in Table 3 were selected for each FMA item for later scoring based on logical rules.

All these rules were based on an IF-ElSEIF-ELSE logic model, as shown in Figure 3B. There was an additional prerequisite for FMA items 14, 17, and 33 (marked gray). For 14 and 17, the prerequisite was whether the subject could move to the initial position, and for 33, the prerequisite was whether the subject could point to the nose with the paretic limb. With regard to item 24 and 25, there was such a logic model for scoring each finger, and finally, the item score was obtained by another model with the total score of four fingers as input. Detailed rules for each item are provided in the Appendix B. The variable with the subscript *p* represents the feature of the paretic side, with the subscript *h* represents the less-affected side, and with the subscript *N* represents the normal reference, which is a constant calculated from the mean value of all subjects’ less-affected side features. Notably, the threshold $\lambda_{1}$ and $\lambda_{2}$ in each rule were set as 1/3 and 2/3, respectively, achieving the effect of three equal divisions, which were approved by experienced clinicians.

### 2.4. Data Analysis

The following metrics were calculated in this study. By default, the Leave-One-Out Cross-Validation (LOOCV) method [37] was used for performance evaluation of the RF segment, and the average less-affected-side kinematic features of all subjects were used as the standard references for the RB segment. The LOOCV method uses one subject as the test set and all other subjects as the training set and iterates this step *n* times (*n* is the sample size). Then offline scores for each item of each person could be obtained.

1. Total scores: Using Pearson’s correlation coefficient, the correlation between the system and therapist scores was investigated. In addition, in order to further prove the system value in practical applications, a simulated online test was also implemented. The 20 participating patients were first ranked in ascending order according to their FMA-UE scores. In order to ensure the involvement of different motor function levels, every other subject was selected to construct a ten-patient training set, and the remaining ten patients made up the test set. For convenience, the result predicted by the automated system was abbreviated as S_FMA and that evaluated by the therapist was abbreviated as T_FMA.
2. Single FMA items: To evaluate the scoring accuracy of the proposed system, both the prediction accuracy and mean absolute error (MAE) of each item were calculated, using scores obtained from the therapist as the gold standard. The consistency between all the scores for a total of 600 items (30 items for each of 20 subjects) obtained by these two assessment methods was estimated by linear weighted Cohen’s kappa coefficient. Four additional macro-averaged metrics, including F1-score, sensitivity, specificity, and precision, were also calculated according to the confusion matrix.

All statistical analyses were performed via SPSS (IBM, Chicago, IL, USA).

## 3. Results

### 3.1. Participants

A total of 20 patients with stroke participated in this study. Table 4 presents the detailed characteristics of the population.

### 3.2. Performance Evaluation on Total Scores

**Figure 4:**
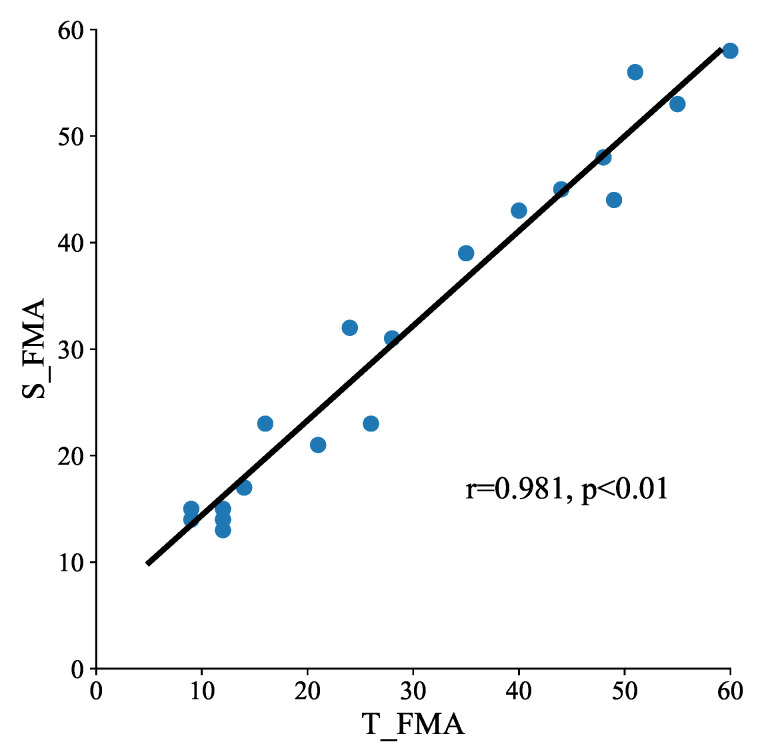
The correlation between the summed scores of the 30 items obtained by the automated system and by a therapist (*n* = 20).

Correlation between S_FMA and T_FMA was particularly high (r = 0.981, *p* < 0.01) (Figure 4), indicating that the system has a strong ability to produce FMA-UE scores consistent with the therapist.

**Figure 5:**
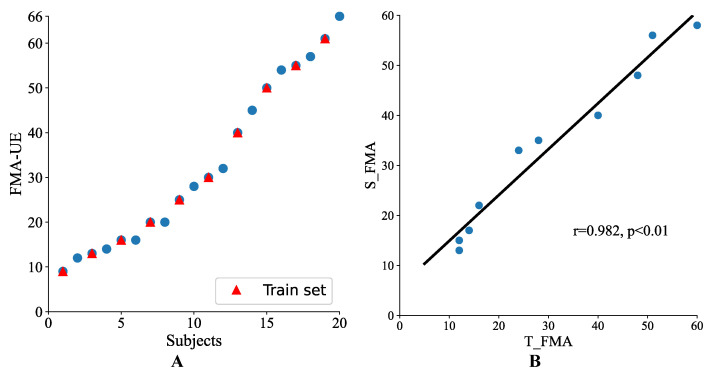
(**A**) The FMA-UE scores of 20 subjects. The subjects with red triangle markers were regarded as the training set in the simulated online test, and other subjects were used as the test set. (**B**) Simulated online test results, the correlation between the total scores of the 30 items obtained by the automated system and by a therapist in the test set (*n* = 10).

In order to further verify the system value in practical applications, online testing was simulated. The FMA-UE scores of the 20 participating patients in this study are displayed in Figure 5A in ascending order, which are evenly distributed. As shown in the Figure 5B, Pearson’s correlation coefficient between S_FMA and T_FMA (r = 0.982, *p* < 0.01) of the simulated online test was equal to the offline test.

### 3.3. Performance Evaluation on Single FMA Items

**Figure 6:**
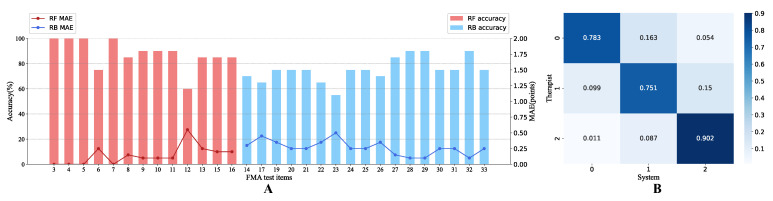
The results of single FMA items. (**A**) Prediction accuracy (%) and mean absolute error (MAE) (points) of each automated FMA item, where the red part denotes the items of the Shoulder/Elbow category scored by random forest classification, and the blue part denotes those of the other three parts scored by rule-based logic methods. The bar graph is the accuracy, while the line graph is the MAE. (**B**) A confusion matrix showing single item scores assigned by a therapist versus estimated by the automated system, with 600 items in total (30 items for each of 20 subjects).

Figure 6A depicts the performance of single FMA items in two indicators: classification accuracy and MAE. Due to different scoring methods and accuracy evaluation approaches, all items were divided into two segments to show the results: the Shoulder/Elbow part and the other three parts, distinguished by two different colors. Item 3, 4, 5, and 7 had the highest accuracy (100%), and item 12 and 23 had the lowest accuracy (60% and 55%, respectively) among each segment. The maximum error (0.55 points) appeared in item 12, which was still far less than the resolution of the scale: 1 point.

Figure 6B shows the confusion matrix of FMA scores assigned by the therapist versus scores estimated by the automated system. The accuracy for score 0 (78.3%) and 2 (90.2%) was higher than that for score 1 (75.1%), implying that the system performed better in extreme cases. Meanwhile, prediction errors mainly occurred in misjudgment of score 0 as 1 (16.3%) and misjudgment of score 1 as 2 (15%), that is, the system tended to overestimate results when it deviated from the therapist assessment. Cohen’s kappa coefficient was 0.757, demonstrating a substantial agreement between the two scoring manners.

The mean accuracy, macro-averaged F1-score, precision, sensitivity, and specificity, and MAE for each segment and for all the selected 30 FMA items are shown in Table 5. The average accuracy of the RF classification segment was as high as 88.08% and of all 30 items was over 80.83%. The macro-averaged F1-score, precision, sensitivity, and specificity of all 30 items were 80.97%, 81.11%, 81.22%, and 90.40%, respectively, and there is no significant deviation between precision and sensitivity. It suggests that the system was well behaved under all these performance measures. The average MAE of all 30 items was 0.21.

## 4. Discussion

In the present study, we proposed a complete automated FMA system to independently assess upper limb motor function in stroke patients and performed preliminary validation. On the whole, the total scores of the system were highly linearly correlated with that of the therapist, which was very close to 1. In terms of single FMA items scores, there was a considerable agreement between these two assessment methods (System and Therapist), as shown in Cohen’s kappa coefficient. Meanwhile, detailed scores for each FMA item were also available, though some still had room for improvement in accuracy.

For the Shoulder/Elbow part using RF classification, the accuracy of each item could exceed 75% except for item 12 (hand to lumbar spine). For the other three parts using rule-based logical classification, the accuracy of each item exceeded 65% except for item 23 (wrist circumduction). The highest accuracy 100% occurred in item 3, 4, 5 and 7, which were parsed from one motion. This reveals that the system can successfully evaluate multiple aspects in a comprehensive motion with synergies. Apart from the latent defects in data processing and scoring methods, the motion implementation was also an essential factor contributing to the poor results of item 12 and 23. Item 12 has a short action stroke, enhancing the difficulty of motion distinction. Besides, some patients who could complete this motion well hid their entire forearms behind their backs, causing the misidentification of key skeletal points. For item 23, it was found that some subjects had difficulty understanding and performing the wrist circumduction movement. Even when using the less-affected side, they might perform compensatory movements. This could mislead both the therapist and the system.

In the previous studies of automated FMA, Kim et al. [17] only used Kinect to automate 13 FMA with an average accuracy below 80%. Bai et al. [18] combined Kinect and one posture sensor to automate 15 items and the accuracy rates range from 73% to 92.7%. Song el al. [38] used a cellphone as a wearable sensor and developed a cellphone-based system for 20 items, whose average accuracy is 85%. However, they only recruited 10 patients, and patients with scores between 30–50 were missing. Lee et al. [20] combined Kinect and FSRs to increase the automated items to 25, achieving an average accuracy of 92% in merely 9 subjects. Compared with them, the proposed system had comparable results with more automated items validated in a larger number of subjects.

The single item result of MAE was highly negatively correlated with that of accuracy (r = −0.976, *p* < 0.01 for RF classification, r = −0.961, *p* < 0.01 for RB classification). The two indicators were almost identical, and most items with the same accuracy rate also had the same error. One possible reason is that the number of cross-level misclassification samples for this system was small. In the confusion matrix of Figure 6B, only 5.4% of 0 points were mistakenly predicted as 2 points, and 1.1% of 2 points were mistakenly predicted as 0 points. This additionally indicates that the system performs well under the comprehensive evaluation of these two indicators. In terms of the accuracy in 600 items, the order from high to low was score 2, 0, and 1. The characteristics of extreme levels tended to be more pronounced and easier to classify accurately. In contrast, the boundaries between median and extreme levels could be ambiguous, thus resulting in a worse result. Additionally, system errors generally occurred in evaluating a level higher than the therapist, which should be paid attention to when patients or physicians utilize the automated system results.

Furthermore, the satisfactory simulated online test result further proved the accuracy and reliability of the system.It also implied that the proposed method could be promoted and applied in practice without difficulty, especially no additional experiments or prior knowledge was needed for the parameter determination.

Compared with other automated assessment systems based on wearable sensors (such as IMUs) and EMG, the proposed system, mainly relying on RGBD cameras, still has limitations. Wearable sensors allow for more accurate motion data collection without occlusion issues. More importantly, their use is not limited by the time and location, i.e., by applying wearable sensors continuous assessment of activities of daily living [39] and assessment performed at the bedside or in bed can be possible [40]. Unlike kinematic data, EMG can be utilized to analyze the neuromuscular differences under different motor functions from a more fundamental perspective, which cannot be obtained by other sensors. Nevertheless, the preliminary validation of the system is promising, and the assessment results are even better [11,15]. In addition, the proposed system can cover a wider assessment scope, and there is no need to calibrate and wear in advance. To conclude, it is more automated, convenient, and low cost.

By promoting strengths and avoiding or compensating for weaknesses, the proposed assessment system can be improved in the following aspects in the future. First, the accuracy of some single items is kind of unsatisfactory, which resulted in the system tending to overestimate patients’ motor function. In addition to optimizing data processing methods, refining the instructions and adjusting evaluation paradigms of this automated system may also help improve results. Second, the feasibility of the system has only been preliminarily verified in a small number of subjects. The test-retest reliability will subsequently be measured with more patients participation. Moreover, although the sensors used are all non-wearable sensors, the number of sensor types is relatively large (three types of sensors including RealSense, Leap Motion, and FSRs). This automated system, which is preferably arranged in a separate room, is more suitable for hospitals, local clinics, or communities. There is still room for improvement in its convenience. Future work will focus on using RGB cameras alone to simplify the proposed system. In this way, patients can use a computer or a smartphone with cameras for evaluation at home.

## 5. Conclusions

This paper proposed an automated FMA system combining software and hardware, which is suitable for use in hospitals and communities. A high correlation coefficient of 0.981 between system and therapist and an average accuracy of over 80% for single FMA items were achieved, with 20 patients participating. It demonstrates that this system can supplement and has the potential to replace the manual evaluation of the therapist. Compared to previous studies, the completeness of the system eliminates the need for therapists to perform complementary assessment items. This significantly saves their time and reduces their workload, allowing them to focus on rehabilitation training.

## Figures and Tables

**Table 1:** Target FMA items and Grouping ^1^.

Category ^2^	Motion ^3^	FMA Item	Sensors ^4^
Reflex activity		1. Flexors	
		2. Extensors	
**Shoulder/Elbow** Volitional movement within synergies	I	3. Shoulder elevation	RS
		4. Shoulder retraction	RS
		5. Shoulder abduction(90${}^{\circ }$)	RS
		6. Shoulder external rotation	RS
		7. Elbow flexion	RS
		8. Forearm supination	RS
		9. Shoulder adduction/internal rotation	RS
		10. Elbow extension	RS
		11. Forearm pronation	RS
**Shoulder/Elbow** Volitional movement mixing synergies	II	12. Hand to lumbar spine	RS
	III	13. Shoulder flexion 0∼90${}^{\circ }$	RS
**Shoulder/Elbow** Volitional movement with little or no synergy	V	15. Shoulder abduction 0∼90${}^{\circ }$	RS
	III	16. Shoulder flexion 90∼180${}^{\circ }$	RS
Normal reflex activity		18. Biceps, triceps, finger flexors	
**Wrist/Hand**	IV + VI${}_{90}$	14. Forearm pronation-supination with elbow 90${}^{\circ }$	RS + LM
	III + VI${}_{0}$	17. Forearm pronation-supination with elbow 0${}^{\circ }$	RS + LM
	VII${}_{90}$	19. Stability at $15^{\circ }$ dorsiflexion with elbow 90${}^{\circ }$	LM
		20. Repeated dorsiflexion/volar flexion with elbow 90${}^{\circ }$	LM
	VII${}_{0}$	21. Stability at $15^{\circ }$ dorsiflexion with elbow 0${}^{\circ }$	LM
		22. Repeated dorsiflexion/volar flexion with elbow 0${}^{\circ }$	LM
	VIII	23. Circumduction	LM
	IX	24. Mass flexion	LM
		25. Mass extension	LM
**Grasp**	X	26. Hook grasp	FSRs
	XI	27. Thumb adduction	FSRs
	XII	28. Pincer grasp	FSRs
	XIII	29. Cylinder grasp	FSRs
	XIV	30. Sphere grasp	FSRs
**Coordination/Speed**	XV	31. Tremor	RS
		32. Dysmetria	RS
		33. Time	RS

^1^ The gray part indicates the items not included in the automated system, the red part indicates the items scored using random forest classification, and the blue part indicates the items scored using rule-based logic classification. ^2^ The FMA items involved in the automated system were grouped into four categories (bolded) based on the execution actions and evaluation methods. ^3^ 30 FMA items were implemented in this automated system with 17 motion tasks, with multiple FMA items corresponding to one motion, or one FMA item split into two motions in some cases. ^4^ RS means RealSense, LM means Leap Motion, and FSRs means Force Sensitive Resistors.

**Table 2:** Raw data information.

Data Source	RealSense D435	Leap Motion	Force Sensitive Resistors
**Sampling frequency**	30 fps	200 fps	10 Hz
**Experimental protocol**	Perform motions I–V, XV	Perform motions VI-IX	Grasp 5 specified tools
**Original data format**	Color and depth images	Hand tracking data	Voltage and force
**Features** ^1^	3D coordinates of joints (f×18×3)	Angles (f×a)	Force (f×1)
**Sample Size**	20 subjects × 2 repetitions	20 subjects	20 subjects

^1^*f* is the frame number, and a is the angle number.

**Table 3:** Extracted Features for Each FMA Test Item.

Category	FMA Item	Feature Category/Symbol	Feature
**Shoulder/Elbow**	3–17 (except 14,17)	Endpoint	Path length of the endpoint
			Max velocity
			Mean velocity
			Velocity variance
			Spectral arc length
			Dimensionless jerk
		Angle (Shoulder flexion, shoulder adduction, shoulder rotation, elbow flexion)	Range of motion (ROM)
			Max angle
			Min angle
			Max angular velocity
			Mean angular velocity
			Angular velocity variance
		Others	Inter-joint coordination index
			Time
			Max shoulder joint displacement (X, Y, Z)
			Trunk compensation
**Wrist/Hand**	14	$\delta_{max},\Phi_{max}$	Max elbow flexion angle and forearm angle
	17	$\alpha_{max},\Phi_{max}$	Max shoulder flexion angle and forearm angle
	19–22	$\theta_{max},\theta_{min}$	Wrist pitch angle (max, min)
	23	$\theta_{max},\psi_{max}$	Wrist pitch angle and yaw angle (max, min)
	24, 25	$\eta_{i,max},\rho_{max}$	Finger tip anlge and hand grab strength (max, min)
**Grasp**	26–30	$V_{max},F_{max}$	Max voltage and force
**Coordination/Speed**	31	SPARC	Spectral arc length
	32	$d_{r,min}$	Min relative distance between wrist and nose
	33	*T*	Time

**Table 4:** Characteristics of Stroke Subjects.

Index	Age	Sex	Time since Stroke Onset (Month)	Paretic Side	MMSE	Brunnstrom	FMA-UE
P1	54	M	2	Left	28	2	14
P2	70	F	4	Right	29	6	66
P3	51	M	1	Left	30	4	54
P4	61	M	7	Left	28	3	32
P5	43	F	2	Left	29	4	45
P6	70	F	24	Left	27	2	9
P7	69	F	3	Left	29	5	57
P8	58	M	2	Right	30	5	61
P9	58	M	4	Right	28	3	20
P10	54	M	1	Right	28	2	13
P11	73	M	7	Left	24	2	25
P12	68	M	2	Right	28	4	28
P13	33	M	11	Left	28	4	30
P14	56	M	24	Right	29	4	40
P15	74	M	2	Right	27	3	16
P16	61	M	22	Left	27	2	12
P17	70	F	1	Left	28	3	20
P18	58	M	5	Left	28	5	55
P19	51	M	2	Left	27	3	16
P20	47	M	1	Left	28	5	50

**Table 5:** Average results of performance metrics for single items.

Indicators	Shoulder/Elbow Part	The Other Three Parts	All Four Parts
Accuracy (%)	88.08	75.30	80.83
F1-score (%)	86.59	74.58	80.97
Precision (%)	88.70	74.27	81.11
Sensitivity (%)	85.81	75.22	81.22
Specificity (%)	93.77	87.72	90.40
Mean absolute error	0.15	0.26	0.21

**Figure A1:**
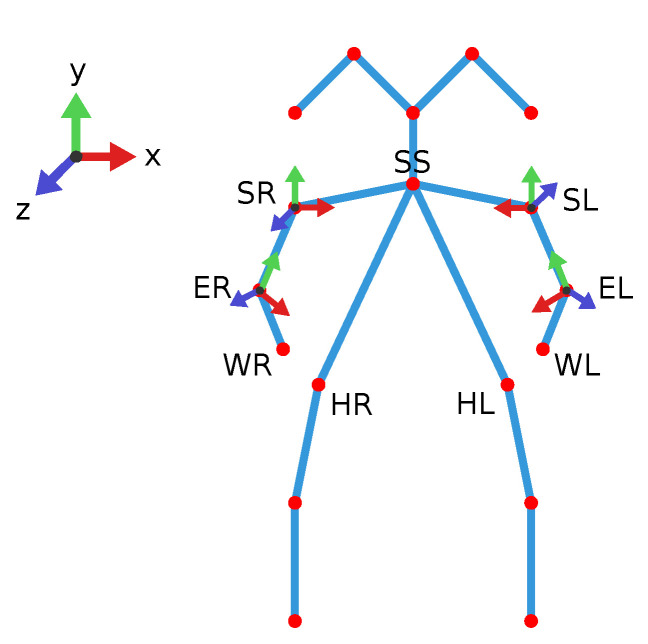
Coordinate systems of the thorax and the humerus segments.

## Data Availability

Data are available on request due to privacy restrictions.

## Appendix A. Detailed Feature Extraction Methods

To construct the kinematic metrics in the endpoint space, the endpoint displacement $d_{i}$ between two frames is calculated first: $d_{i}=∥W_{i}-W_{i-1}∥,i=1,\cdots ,n$ where $W_{i}$ is the Cartesian 3D coordinates of the wrist in the *i*th frame and *n* is the total number of recorded frames. $d_{i}$ multiplies fps is the velocity and the sum is the path length.

The computable joint angles involved in the moving process include: shoulder flexion/extension angle (α), adduction/abduction angle (β), internal/external rotation angle (γ) and elbow flexion/extension angle (δ). The first three angles were defined by Euler angles from the rotation matrix with XZY sequence, which is further elaborated below.

The coordinate systems of the thorax and the humerus segments for both sides from the 18-joint skeleton of RealSense is shown in Figure A1. Take the right side as an example, and the left side is similar.

The definition of the thorax segment coordinate system $C_{1}$ is as follows: SpineMid (SM) is defined as the midpoint of HR and HL (A1). The *y*-axis is the unit vector going from SM to SS (A2), the *z*-axis is the unit vector perpendicular to the the plane formed by *y*-axis and the vector from SR to SM (A3), and the *x*-axis is defined by *y* and *z*-axes to create a right-hand coordinate system (A4).

(A1) $$SM=\frac{1}{2}\left(HR+HL\right)$$

(A2) $$y_{C_{1}}=\frac{SS-SM}{∥SS-SM∥}$$

(A3) $$z_{C_{1}}=\frac{\left(SS-SR\right)\times y_{C_{1}}}{∥\left(SM-SR\right)\times y_{C_{1}}∥}$$

(A4) $$x_{C_{1}}=\frac{y_{C_{1}}\times z_{C_{1}}}{∥y_{C_{1}}\times z_{C_{1}}∥}$$

(A5) $$C_{1}=\left[x_{C_{1}},y_{C_{1}},z_{C_{1}}\right]$$

The definition of the right humerus segment coordinate system $C_{2}$ is as follows: the *y*-axis is the unit vector going from ER to SR (A6), the *z*-axis is the unit-vector perpendicular to the plane formed by *y*-axis and the vector from ER to WR (A7), and the *x*-axis is also defined by *y* and *z*-axes to create a right-hand coordinate system (A8).

(A6) $$y_{C_{2}}=\frac{SR-ER}{∥SR-ER∥}$$

(A7) $$z_{C_{2}}=\frac{\left(WR-ER\right)\times y_{C_{2}}}{∥\left(WR-ER\right)\times y_{C_{2}}∥}$$

(A8) $$x_{C_{2}}=\frac{y_{C_{2}}\times z_{C_{2}}}{∥y_{C_{2}}\times z_{C_{2}}∥}$$

(A9) $$C_{2}=\left[x_{C_{2}},y_{C_{2}},z_{C_{2}}\right]$$

Then the rotation Matrix $R_{2}^{1}$ can be obtained via the parent coordinate system $C_{1}$ and the child coordinate system $C_{2}$ (A10). Shoulder flexion/extension, adduction/abduction, and internal/external rotation angles (α,β,γ in order) are Euler angles from *R* with XZY sequence.

(A10) $$R_{2}^{1}=C_{1}^{-1}C_{2}$$

Additionally, the elbow flexion/extension angle is defined as:

(A11) $$\delta =\frac{\vec{SE}\cdot \vec{EW}}{∥SE∥∥EW∥}$$

where $\vec{SE}=SR-ER\;\mathrm{or}\;SL-EL$, and $\vec{EW}=ER-WR\;\mathrm{or}\;EL-WL$.

The minimum relative distance between wrist and nose is defined as:

(A12) $$d_{r,min}=\min\left(\frac{∥NW∥}{∥NK∥}\right)$$

where NW=N−WR,NK=N−KR or NW=N−WL,NK=N−KL.

## Appendix B. Scoring Rules

The scoring rules are listed in Table A1 and Table A2.

**Table A1:** Logic Classification Rules.

Category	FMA Item	Rule ^1^	Score ^2^
Wrist/Hand (Rule2 ^3^)	14	If Δ	0
		Else If $\Phi_{1}$	0
		Else if $\Phi_{2}$	2
		Else	1
	17	If *A*	0
		Else If $\Phi_{1}$	0
		Else if $\Phi_{2}$	2
		Else	1
	19, 21	If $\Theta_{1}$	0
		Else if $\Theta_{2}$	2
		Else	1
	20, 22	If ($\Theta_{1}$ AND $\Theta_{3}$) OR $\Theta_{5}$	0
		Else if $\Theta_{2}$ AND $\Theta_{4}$ AND $\Theta_{6}$	2
		Else	1
	23	If $\Theta_{5}$ AND $\Psi_{1}$	0
		Else if $\Theta_{6}$ OR $\Theta_{2}$	2
		Else	1
	24	If $H_{1i}$ $s_{i}=0$	
		Else if $H_{2i}$ AND $H_{6i}$ $s_{i}=2$	
		Else $s_{i}=1$	
		If $\sum_{i=2}^{5}s_{i}<8\lambda_{1}$	0
		Else if $\sum_{i=2}^{5}s_{i}>8\lambda_{2}$ AND $P_{1}$	2
		Else	1
	25	If $H_{3i}$ OR $H_{5i}$ $s_{i}=0$	
		Else if $H_{4i}$ $s_{i}=2$	
		Else $s_{i}=1$	
		If $\sum_{i=2}^{5}s_{i}<8\lambda_{1}$	0
		Else if $\sum_{i=2}^{5}s_{i}>8\lambda_{2}$ AND $P_{2}$	2
		Else	1
Grasp (Rule3)	26–30	If $F_{max,p}=0$	
		If $V_{max,p}=0$	0
		Else	1
		Else if $F_{max,p}>\lambda_{2}F_{max,h}$	2
		Else	1
Coordination /Speed (Rule1)	31	If $\frac{SPARC_{N}}{SPARC_{p}}<\lambda_{1}$	0
		Else if $\frac{SPARC_{N}}{SPARC_{p}}>\lambda_{2}$	2
		Else	1
	32	If $d_{r,min,h}>\lambda_{2}$	0
		Else if $d_{r,min,h}<\lambda_{1}$	2
		Else	1
	33	If $d_{r,min,p}>\lambda_{2}$	0
		Else If $T_{p}-T_{h}>6$	0
		Else if $\left|T_{p}-T_{h}\right|<2$	2
		Else	1

^1^ λ_1_ = 1/3, λ_2_ = 2/3. 2 Score 0 = cannot perform, 1 = partially completed, 2 = fully completed. ^3^ The meanings of symbols in Rule2 are explained in Table A2.

**Table A2:** Symbol meanings in Rule2.

Symbol	Logic Operation ^1^
*A*	$\alpha_{max,p}<30^{\circ }$
Δ	$\delta_{max,p}<90^{\circ }$
$\Phi_{1}$	$\phi_{max,p}<\lambda_{1}\phi_{max,N}$
$\Phi_{2}$	$\phi_{max,p}>\lambda_{2}\phi_{max,N}$
$\Theta_{1}$	$\theta_{max,p}<\lambda_{1}\theta_{max,N}$
$\Theta_{2}$	$\theta_{max,p}>\lambda_{2}\theta_{max,N}$
$\Theta_{3}$	$-\theta_{min,p}<-\lambda_{1}\theta_{min,N}$
$\Theta_{4}$	$-\theta_{min,p}>-\lambda_{2}\theta_{min,N}$
$\Theta_{5}$	$\left(\theta_{max,p}-\theta_{min,p}\right)<\lambda 1\left(\theta_{max,N}-\theta_{max,N}\right)$
$\Theta_{6}$	$\left(\theta_{max,p}-\theta_{min,p}\right)>\lambda 2\left(\theta_{max,N}-\theta_{max,N}\right)$
$\Psi_{1}$	$\left(\psi_{max,p}-\psi_{min,p}\right)<\lambda 1\left(\psi_{max,N}-\psi_{max,N}\right)$
$\Psi_{2}$	$\left(\psi_{max,p}-\psi_{min,p}\right)>\lambda 2\left(\psi_{max,N}-\psi_{max,N}\right)$
$H_{1i}$	$\eta_{min,pi}>\frac{\eta_{min,Ni}}{\lambda_{1}}$
$H_{2i}$	$\eta_{min,pi}<\frac{\eta_{min,Ni}}{\lambda_{2}}$
$H_{3i}$	$\eta_{max,pi}<\lambda_{1}\eta_{max,Ni}$
$H_{4i}$	$\eta_{max,pi}>\lambda_{2}\eta_{max,Ni}$
$H_{5i}$	$\eta_{max,pi}-\eta_{min,pi}<\lambda_{1}\left(\eta_{max,Ni}-\eta_{min,Ni}\right)$
$H_{6i}$	$\eta_{max,pi}-\eta_{min,pi}>\lambda_{2}\left(\eta_{max,Ni}-\eta_{min,Ni}\right)$
$P_{1}$	$\rho_{max,p}==1$
$P_{2}$	$\rho_{min,p}==0$

^1^ λ_1_ = 1/3, λ_2_ = 2/3.
